# New Cell Lines Derived from European Tick Species

**DOI:** 10.3390/microorganisms10061086

**Published:** 2022-05-25

**Authors:** Lesley Bell-Sakyi, Catherine S. Hartley, Jing Jing Khoo, Jan Hendrik Forth, Ana M. Palomar, Benjamin L. Makepeace

**Affiliations:** 1 Department of Infection Biology and Microbiomes, Institute of Infection, Veterinary and Ecological Sciences, University of Liverpool, 146 Brownlow Hill, Liverpool L3 5RF, UK; catherine.hartley@liverpool.ac.uk (C.S.H.); Jing.Jing.Khoo@liverpool.ac.uk (J.J.K.); blm1@liverpool.ac.uk (B.L.M.); 2 Robert Koch-Institut, Nordufer 20, 13353 Berlin, Germany; Forthj@rki.de; 3 Friedrich-Loeffler-Institut, Federal Research Institute for Animal Health, Südufer 10, 17493 Greifswald-Insel Riems, Germany; 4 Centre of Rickettsiosis and Arthropod-Borne Diseases, Hospital Universitario San Pedro-CIBIR, 26006 Logroño, La Rioja, Spain; ampalomar@riojasalud.es

**Keywords:** tick cell line, vector, *Argas reflexus*, *Dermacentor reticulatus*, *Hyalomma lusitanicum*, *Hyalomma scupense*, *Ixodes ricinus*, *Rhipicephalus bursa*

## Abstract

Tick cell lines are important tools for research on ticks and the pathogens they transmit. Here, we report the establishment of ten new cell lines from European ticks of the genera *Argas*, *Dermacentor*, *Hyalomma*, *Ixodes* and *Rhipicephalus* originating from Germany and Spain. For each cell line, the method used to generate the primary culture, a morphological description of the cells and species confirmation by sequencing of the partial 16S rRNA gene are presented. Further molecular analysis of the two new *Ixodes ricinus* cell lines and three existing cell lines of the same species revealed genetic variation between cell lines derived from ticks collected in the same or nearby locations. Collectively, these new cell lines will support research into a wide range of viral, bacterial and protozoal tick-borne diseases prevalent in Europe.

## 1. Introduction

Ticks are vectors of multiple viral, bacterial and protozoan pathogens of medical and veterinary importance, including tick-borne encephalitis virus (TBEV), Crimean-Congo hemorrhagic fever virus (CCHFV) and multiple species of the genera *Anaplasma*, *Babesia*, *Borrelia*, *Ehrlichia* and *Rickettsia* [1]. Within Europe, the major vector of both human and animal diseases is the ixodid (hard) tick *Ixodes ricinus*, which is distributed widely throughout the continent [2,3]. Amongst the approximately 66 tick species reported from the Western Palearctic, including Europe [4], other important vectors include *Ixodes persulcatus* in Eastern Europe, *Dermacentor* spp. in central and southern (and increasingly northern) Europe, and *Hyalomma* and *Rhipicephalus* spp. in southern Europe.

Tick cell lines form an increasingly valuable part of the toolkit for research on the biology and control of ticks and tick-borne pathogens [5,6,7,8,9,10]. However, historically, much of the cell culture-based research has utilised cell lines derived from non-European ticks, such as the tropical species *Rhipicephalus appendiculatus* and *Rhipicephalus microplus*, and the North American species *Ixodes scapularis*. Prior to the start of the present study, cell lines were only available from two European tick species: the *I. ricinus* lines IRE11 [11], IRE/CTVM19 and IRE/CTVM20 [12] and the *Rhipicephalus sanguineus* line RSE/PILS35 [13].

Part of the remit of the Tick Cell Biobank [5] is to generate novel cell lines from ticks and other arthropods, that are then made available as a resource for the international research community. Here, we describe the establishment of new continuous cell lines from six species of European ticks: the argasid (soft) tick *Argas reflexus* and the ixodid (hard) ticks *Dermacentor reticulatus*, *Hyalomma lusitanicum*, *Hyalomma scupense*, *I. ricinus* and *Rhipicephalus bursa*. Species origin and absence of contaminating bacteria were confirmed by molecular analysis. This study follows and expands the preliminary report of the *A. reflexus* cell line and primary cultures derived from the two *Hyalomma* spp. and *R. bursa* [5].

## 2. Materials and Methods

### 2.1. Ticks

Engorged female ticks were reared in a laboratory colony at the Friedrich-Loeffler-Institut (FLI) derived from individuals originally collected in Berlin (*A. reflexus*), submitted by a member of the public to a screening programme in Germany (*D. reticulatus*), or collected from mammalian hosts in the field, as detailed in Table 1. Field collections in Spain were performed during routine veterinary examinations of cattle with the owners’ consent (La Rioja) or from culled deer (Castilla-La Mancha).

The engorged female ticks were transferred to the Tick Cell Biobank where they were surface-sterilised in a horizontal laminar flow cabinet prior to the start of oviposition. Briefly, the ticks were immersed in 0.1% benzalkonium chloride for 5 min, 70% ethanol for 1 min and two changes of sterile deionised water, dried on sterile filter paper and transferred individually to sterile 35 mm plastic Petri dishes and incubated at 100% humidity in a sealed plastic box at room temperature (*A. reflexus*) or 28 °C (all other species). The ticks were examined daily for commencement of oviposition and subsequent embryonic development.

### 2.2. Preparation and Monitoring of Primary Tick Cell Cultures

When the rectal sac of developing embryos was visible as a white spot inside the eggs, the female tick was removed from the Petri dish and the entire egg batch was immersed in 70% ethanol for 1 min, followed by two rinses in Hanks balanced salt solution (HBSS). Using the flattened end of a glass rod, the eggshells were then gently crushed in HBSS, or complete culture medium if the egg batch comprised <100 eggs, to release the embryonic tissues. The resultant suspension was either transferred directly to a flat-sided culture tube (Nunc, Thermo-Fisher, Loughborough, UK) or, for large egg batches, centrifuged at 200× *g* for 5 min, the supernate was removed, the tissue pellet was resuspended in complete culture medium and transferred to one or more flat-sided tubes. The sealed tubes were incubated flat, or at an angle of ~15° for very small amounts of tissues, at 28 °C in ambient air in a dry incubator.

Four different complete culture media were used singly or in combination; all were supplemented with 2 mM L-glutamine, 100 units/mL penicillin and 100 µg/mL streptomycin, and all were prepared freshly each week. All components were sourced from Invitrogen (Thermo Fisher, Loughborough, UK) or Sigma (Sigma Aldrich, Gillingham, UK), except where indicated. Complete L-15 comprised L-15 (Leibovitz) medium supplemented with 10% tryptose phosphate broth (TPB) and 20% foetal bovine serum (FBS); H-Lac comprised HBSS supplemented with 0.5% lactalbumin hydrolysate and 20% FBS; L-15/MEM comprised equal volumes of L-15 (Leibovitz) medium and Minimal Essential Medium with Hanks salts supplemented with 10% TPB and 20% FBS; L-15B comprised L-15B medium [15] supplemented with 10% TPB, 10% FBS and 0.1% bovine lipoprotein concentrate (MP Biomedicals, Thermo Fisher, Loughborough, UK).

All cultures were examined weekly by inverted microscope prior to changing medium. Medium changes involved removal and replacement of between half and three-quarters of the medium volume. Subcultures, only initiated when significant cell multiplication was observed, were performed by adding an equal volume (2.2–2.5 mL) of fresh medium to the primary culture, resuspending the cells and tissue clumps by pipetting, and transferring half of the resultant suspension to a new flat-sided tube. The other half of the culture was retained in the parent tube. In some cases, two primary cultures were combined in one of the parent tubes after 6–44 months, to increase the total amount of cells and tissues in an attempt to stimulate cell growth.

### 2.3. Generation and Cryopreservation of Tick Cell Lines

Subculture series were initiated from primary cultures exhibiting significant cell growth (most or all of the flat surface of the culture tube covered with cells and/or tissue clumps, patches of proliferating cells at multiple sites in the tube). In some cases, cell growth was stimulated by resuspending some of the cells and tissue clumps and allowing them to reattach. Subcultures were performed by adding an equal volume of fresh complete culture medium to the culture tube, resuspending the cells and tissues by pipetting, and transferring half of the resultant suspension to a new daughter tube.

When sufficient numbers of daughter tubes had been generated, the contents of 3–4 tubes were pooled and centrifuged at 200× *g* for 5 min, the supernate was discarded, the cell pellet was resuspended in 1.5 mL of ice-cold complete medium, an equal volume of ice-cold complete medium with 20% dimethyl sulphoxide was added and the cell suspension was immediately divided in 1 mL aliquots into three ice-cold cryovials. The cryovials were frozen rapidly in dry ice and transferred within 30 min to the vapour phase of a liquid nitrogen refrigerator. Cryopreserved cells were resuscitated by rapidly thawing the cryovial in a 37 °C water bath; the contents were immediately added to 9 mL complete medium at room temperature and centrifuged at 200× *g* for 5 min. The cell pellet was resuspended in 2.2 mL of complete medium, transferred to a culture tube and incubated at 28 °C. When cryopreserving cells grown in culture medium with an FBS concentration below 20%, the FBS concentration in the freezing and thawing media was increased to 20%.

All cell lines generated in the present study are available under Material Transfer Agreement from the Tick Cell Biobank.

### 2.4. Confirmation of Species Origin and Screening for Contaminating Microorganisms

DNA was extracted from resuspended tick cells using a DNeasy Blood and Tissue kit (Qiagen, Hilden, Germany), following the manufacturer’s instructions for cultured cells. To confirm the species origin, a 456 bp fragment of the tick 16S rRNA gene was amplified as described previously [16] using primer pairs 16S + 1 and 16S − 1. A pan-bacterial PCR targeting a ~1500 bp fragment of the bacterial 16S rRNA gene with the primer pair fD1 and rP2 [17] was used to screen the cell lines for presence of endosymbiotic or contaminating bacteria; *Ehrlichia ruminantium* DNA was used as a positive control. PCR amplicons were visualised by agarose gel electrophoresis, positive PCR products were purified using a Qiagen PCR Purification Kit (Qiagen, Manchester, UK) or a PureLink™ Quick Gel Extraction and PCR Purification Combo Kit (Invitrogen, Loughborough, UK) following the manufacturer’s instructions, and Sanger-sequenced in both directions (Source BioScience, Nottingham, UK). Randomly-selected cultures of each cell line were screened for *Mycoplasma* spp. using two commercial tests, the Mycoalert Mycoplasma Detection Kit (Lonza, Fisher Scientific, Loughborough, UK) and the PCR Mycoplasma Test Kit (Promocell, VWR, Lutterworth, UK), following the manufacturers’ instructions.

Further analysis of the species origin of the cell lines derived from eggs laid by ticks identified morphologically as *I. ricinus* was conducted using PCRs targeting the tick *coxI* [18] and *trospA* [19] genes following the published protocols. DNA extracted from additional *I. ricinus* cell lines IRE/CTVM19, IRE/CTVM20 and IRE11 [11,12] was also tested using these PCR assays and the tick 16S rRNA PCR.

### 2.5. Sequence and Phylogenetic Analyses

Pair-end sequences obtained from PCR amplicons were assembled to produce corrected consensus sequences using 4Peaks (Nucleobytes B.V., Aalsmeer, The Netherlands) and the MUSCLE alignment in AliView [20]. The sequences were then compared with published sequences using BLASTN against the non-redundant database at the National Center for Biotechnology Information (NCBI) GenBank. Multiple sequence alignment was performed using MUSCLE in AliView [20] and visualised using Jalview v2 [21]. Maximum likelihood phylogeny of the partial 16s rRNA, *coxI* and *trospA* gene sequences were generated using PhyML 3.0 [22] with 500 bootstrap replicates. The best-fit model of nucleotide substitution was estimated by Akaike information criterion (AIC) as implemented in jModelTest 2.1.10 [23]. The models selected were Hasegawa–Kishino–Yano (HKY) for all three genes. The resulting trees were imported into FigTree v1.4.4 (http://tree.bio.ed.ac.uk/software/figtree/) for visualisation and the final figures were produced using Inkscape 1.1.2 (https://inkscape.org).

Sequences generated during this study that were not identical to already-published sequences were deposited in GenBank under accession numbers ON366971–ON366983 for tick 16S rRNA sequences, ON367517–ON367521 for *coxI* sequences and ON375792–ON375796 for *trospA* sequences.

## 3. Results

During the course of this study, ten cell lines were generated from European species of the genera *Argas*, *Dermacentor*, *Hyalomma, Ixodes* and *Rhipicephalus* originating from Germany and Spain (Table 2). The cell lines were considered established at the time point when they could be successfully cryopreserved and resuscitated. All the cell lines were found to be free of contaminating bacteria by bacterial 16S rRNA PCR and screening of culture supernate for *Mycoplasma*, and there was no microscopic evidence of contaminating microorganisms in any of the lines.

### 3.1. Argas reflexus

**Figure 1:**
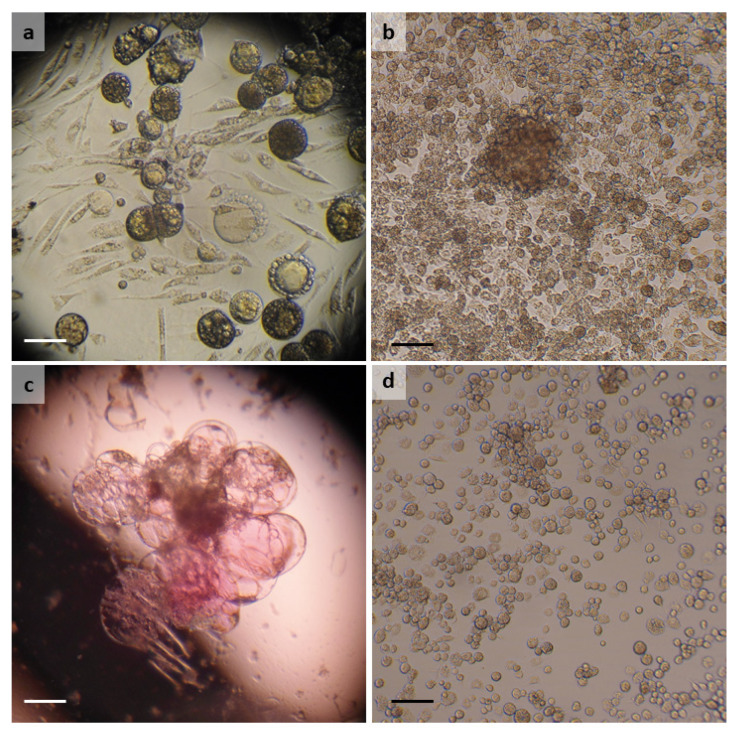
*Argas reflexus* and *Dermacentor reticulatus* cell lines. (**a**) *A. reflexus* primary culture 4 months post initiation showing large floating vacuolated cells, smaller attached spindle-shaped cells and small round haemocyte-like cells; live, inverted microscope; (**b**) *A. reflexus* cell line ARE/LULS41 at passage 8, 5 years post initiation; live, inverted microscope; (**c**) cluster of floating vacuolated cells in *D. reticulatus* primary culture, 15 months post initiation, showing pink colouring of some vacuoles; (**d**) *D. reticulatus* cell line DRE/LULS60 at passage 7, 3 years post initiation. Live, inverted microscope, scale bars = 100 µm.

A single *A. reflexus* female laid ten eggs approximately ten weeks after feeding. Nine of the eggs were used when less than 2 weeks old to set up a primary culture in 0.5 mL L-15 medium in a slanted tube. For the first few weeks, no medium was removed and a single drop of fresh medium was added fortnightly. After 3 months, medium changes were performed weekly by removal and replacement of half the volume. At first, the culture comprised mainly a variety of large, round floating cells containing multiple vacuoles and smaller, attached, spindle-shaped cells (Figure 1a). After 4 months, the large cells began to be replaced by a population of smaller, granular, haemocyte-like cells which proliferated. The first, unsuccessful subculture was carried out at 7 months; a second attempt at 11 months was successful, leading to establishment of the cell line ARE/LULS41 (Figure 1b) at 35 months. (Table 2). The ARE/LULS41 cell line reached passage 10 and currently comprises a mixture of small, semi-adherent haemocyte-like cells and larger, less granular rounded cells (Figure 1b). The 16S rRNA gene sequence amplified from ARE/LULS41 DNA was 100% identical (100% query cover) to a sequence obtained from an *A. reflexus* nymph collected in the field in Spain (MW289076 [24]).

### 3.2. Dermacentor reticulatus

A single *D. reticulatus* egg batch was processed in January 2019, ten days after the start of oviposition. The primary culture was initiated in L-15/H-Lac/L-15B medium and initially comprised clumps of embryonic tissues and individual semi-adherent haemocytes, which disappeared within a few weeks. Some of the tissues formed large assemblies of floating vacuoles, the contents of which exhibited a range of pH evidenced by colours varying from yellow (acidic) to pink (alkaline) (Figure 1c). Haemocytes reappeared, accompanied by patches of smaller, attached, rounded cells, after 7 months, and these slowly proliferated. The first subculture was carried out at 15 months, and from passage 2, the cells were maintained in L-15B medium. Cells at passage 3 were successfully cryopreserved 29 months post initiation and the resultant cell line DRE/LULS60, comprising attached, haemocyte-like cells growing singly or in clumps (Figure 1d), reached passage 10 at the time of writing (Table 2). The 16S rRNA gene sequence amplified from DRE/LULS60 DNA was 100% identical (100% query cover) to sequences obtained from *D. reticulatus* ticks from, for example, Poland (MK671590 [25]), Spain (MH645514 [26]) and Germany (JF928493 [27]).

### 3.3. Hyalomma lusitanicum

**Figure 2:**
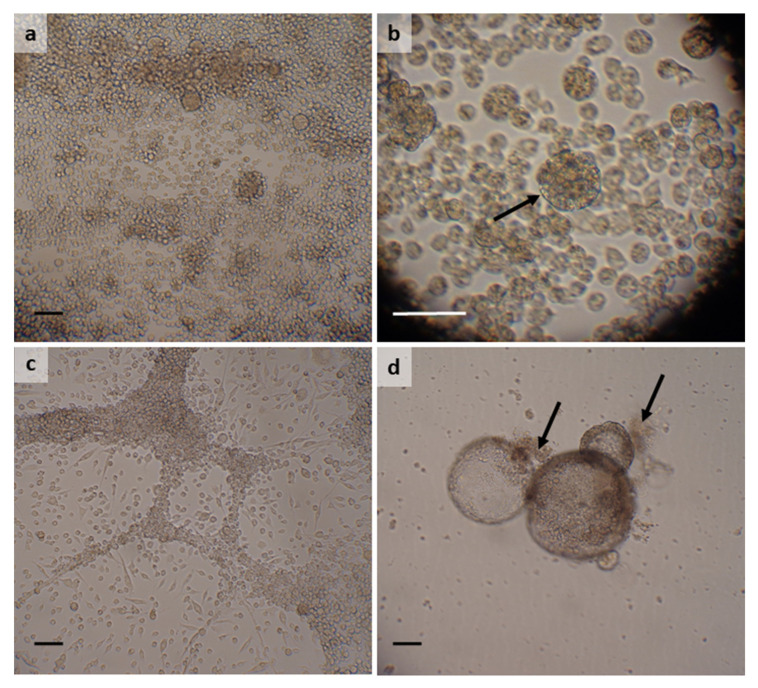
*Hyalomma lusitanicum* and *Hyalomma scupense* cell lines. (**a**) *H. lusitanicum* cell line HLE/LULS42 at passage 2, 8 years after initiation; (**b**) *H. lusitanicum* cell line HLE/LULS48 at passage 6, 8 years after initiation, showing a cell cluster bounded by a membrane (arrow); (**c**) *H. scupense* cell line HSE/LULS51 at passage 9, 6 years after initiation; (**d**) *H. scupense* cell line HSE/LULS59 at passage 9, 6 years after initiation, showing extracellular matrix associated with the multicellular vesicles (arrows). Live, inverted microscope, scale bars = 100 µm.

Three primary cultures were initiated from each of three *H. lusitanicum* egg batches laid by engorged female ticks collected in February (*n* = 2) and May (*n* = 1) 2014 and processed 19–22 days after the start of oviposition. The culture media used initially were L-15, H-Lac, L-15/MEM, L-15/H-Lac, L-15/L-15B and L-15/H-Lac/L-15B. All nine primary cultures survived for at least 25 months, with subculture series initiated from most of them after 8–14 months. Two primary cultures in L-15/H-Lac and L-15/MEM died after 25 and 36 months, respectively, and three were lost to yeast contamination after 41 months. Three of the remaining four primary cultures yielded cell lines: HLE/LULS42 originally in L-15 and now in L-15/L-15B, and HLE/LULS43 and HLE/LULS48 originally in H-Lac and now in L-15/H-Lac/L-15B (Table 2). Establishment of the *H. lusitanicum* cell lines took between 3.5 and 6.25 years; the fourth surviving primary culture, in L-15/H-Lac medium, is also expected to yield a cell line as a subculture series was initiated when significant cell proliferation started after seven years in vitro. All three cell lines comprise attached and floating round, granular cells resembling haemocytes (Figure 2a) and larger, floating clumps of cells in some cases surrounded by a clearly visible peritrophic membrane-like structure (Figure 2b). The 16S rRNA sequences amplified from DNA from HLE/LULS42 (306 bp) and HLE/LULS48 (404 bp), both derived from the same egg batch, were identical to each other. The HLE/LULS48 sequence showed 99.45% similarity (98% query cover) to a *H. lusitanicum* sequence from Portugal (KU130444 [28]) and 99.70% similarity (94% query cover) to a *H. lusitanicum* sequence from Spain (Z97881 [29]). The 16S rRNA sequence obtained from HLE/LULS43, derived from a different egg batch, showed two nucleotide differences from those of the other two *H. lusitanicum* cell lines, but was also 99.50% identical (98% query cover) to the *H. lusitanicum* sequence from Portugal (KU130444) and was 100% identical (96% query cover) to a *H. lusitanicum* sequence from Malta (MK946449 [30]).

### 3.4. Hyalomma scupense

Two or three primary cultures were initiated from each of five *H. scupense* egg batches laid by engorged females collected in February 2016. The eggs were processed 24–35 days after the start of oviposition. Of the twelve resultant primary cultures, two were contaminated with mould and were discarded. The remaining ten cultures were maintained for at least 17 months, at which point two more were lost to yeast contamination. Cells began to grow in several of the primary cultures and subculture series were initiated, leading to establishment of two *H. scupense* cell lines, HSE/LULS51 and HSE/LULS59 in L-15 and L-15/H-Lac, respectively. HSE/LULS51, now at passage 11, comprises small, rounded cells and some areas of cells arranged as sheets or ropes, to which round cells may attach (Figure 2c). In contrast, HSE/LULS59, now at passage 9 and derived from two primary cultures combined after 45 months, comprises floating, spherical and irregularly-shaped multicellular vesicles up to 0.4 mm in diameter, associated with extracellular matrices of heterogeneous material, which also becomes deposited on the surface of the culture tube (Figure 2d). The 16S rRNA sequence amplified from HSE/LULS51 DNA was 100% identical to sequences from *H. scupense* from France (100% query cover, OM736183, OM736184, OM736185) and Russia (97% query cover, KU130468 [28]). The HSE/LULS51 sequence was also 99.51% identical to a sequence from a *H. scupense* tick from Turkey (MW546282) and 99.75% identical to a sequence from a *Hyalomma detritum* tick from China (KC203346 [31]), all with 100% query cover. *H. detritum* was recently reclassified as *H. scupense* [32]. The 16S rRNA sequence amplified from HSE/LULS59 was 99.26–99.49% identical (97–100% query cover) to sequences from *H. scupense* ticks from Pakistan (MN726557 [33] and KU130469 [28]). Interestingly, the sequences from HSE/LULS51 and HSE/LULS59 only shared 98.02% identity (100% query cover), despite being collected from the same geographic area at the same time.

### 3.5. Ixodes ricinus

**Figure 3:**
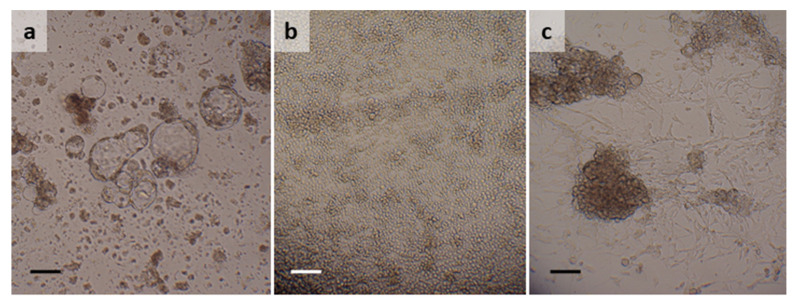
*Ixodes ricinus* and *Rhipicephalus bursa* cell lines. (**a**) *I. ricinus* cell line IRE/LUAP46 at passage 3, 7 years after initiation; (**b**) *I. ricinus* cell line IRE/LULS55 at passage 19, 44 months after initiation; (**c**) *R. bursa* cell line RBE/LULS58 at passage 3, 4 years after initiation. Live, inverted microscope, scale bars = 100 µm.

Two cell lines were generated from eggs laid by ticks that were morphologically identified as *I. ricinus*. The first, IRE/LUAP46, was derived from eggs laid by a tick collected in September 2015 and processed approximately three months later. The primary culture was initiated in L-15 and comprised tissue clumps and a few uncrushed eggs, some of which hatched into larvae that survived for several weeks in the culture medium as shown by the observation of limb movements and midgut peristalsis visible through the larval integument. Although the tissue clumps survived, no appreciable cell growth occurred for the first two years; during the last few months of this period, medium changes were conducted every 2–3 weeks as there was little evidence of metabolism. From 26 months onwards, metabolism resumed and from 29 months floating multicellular vesicles began to appear, accompanied by increased metabolism indicated by a drop in the pH of the medium (from red to orange-yellow colour). Weekly medium changes were resumed at 31 months and the first successful subculture was carried out at 34 months (Table 2). The IRE/LUAP46 cell line, considered established at 45 months, comprises floating multicellular vesicles up to 0.5 mm in diameter (Figure 3a); although during subculture and cryopreservation the vesicles collapse, they reform within 1–2 weeks of disturbance. The 16S rRNA sequence amplified from IRE/LUAP46 DNA was 99.75% identical (100% query cover) to sequences from *I. ricinus* ticks from Poland (MK671589 [25]), Spain (MH645522 [26] and GU074606 [19]) and Portugal (MF370650 [34]) among others.

The second cell line, generated from eggs laid by ticks morphologically identified as *I. ricinus*, IRE/LULS55, was derived from the progeny of three ticks collected in February 2018 and subsequently held at 15 °C instead of 28 °C for five months. Two primary cultures were initiated in L-15/H-Lac and L-15/L-15B from these eggs; as neither culture contained sufficient tissues to support cell line development, they were combined at six months in L-15/H-Lac/L-15B medium. Proliferation of very small round and spindle-shaped cells commenced at 18 months and the first subculture was carried out at 20 months (Table 2). Growth was rapid thereafter, with the IRE/LULS55 cell line becoming established at 29 months and reaching passage 23 at the time of writing. The cell line comprises small, predominantly attached, rounded cells, some of which have the appearance of haemocytes (Figure 3b). The 16S rRNA sequence amplified from IRE/LULS55 DNA was 100% identical (100% query cover) to sequences from *I. ricinus* ticks from The Netherlands and France (GU074607 and GU074618 [19]) and an *Ixodes* sp. tick from the UK (MW727263 [35]). However, the IRE/LULS55 sequence also showed a high identity (99.47–99.51%; 91.38–100% query cover) with ticks described in GenBank as *I. inopinatus* from Tunisia (KM211789 [36] and GU074596 [19]) and Spain (KM211790 [36]). The IRE/LULS55 16S rRNA sequence shared 98.28% identity (100% query cover) with that of IRE/LULS46.

The contrasting results obtained from the 16S rRNA sequence analysis of the two cell lines derived from Spanish ticks morphologically identified as *I. ricinus* prompted further examination of additional gene sequences amplified from these two lines and from DNA extracted from three other *I. ricinus* cell lines: IRE/CTVM19, IRE/CTVM20 and IRE11. The 16S rRNA sequences from IRE/CTVM19 and IRE/CTVM20, both derived from the same pool of four egg batches laid by UK ticks [12,37], were not identical to each other, being, respectively, 99.75% similar (100% query cover) to *I. ricinus* sequences from Spain (MH645519 [26]), Sweden (KX384805 [38]) and Italy (KF197126 [39]) among others, and 100% identical (100% query cover) to *I. ricinus* from Slovakia (GU074588 [19]) and Italy (KF197124 [39]) among others. The 16S rRNA sequence from IRE11, derived from a single egg batch laid by a German tick [11], was 100% identical (100% query cover) to *I. ricinus* sequences from Portugal (MF370649 [34]) and France (GU074610 [19]) among others.

**Figure 4:**
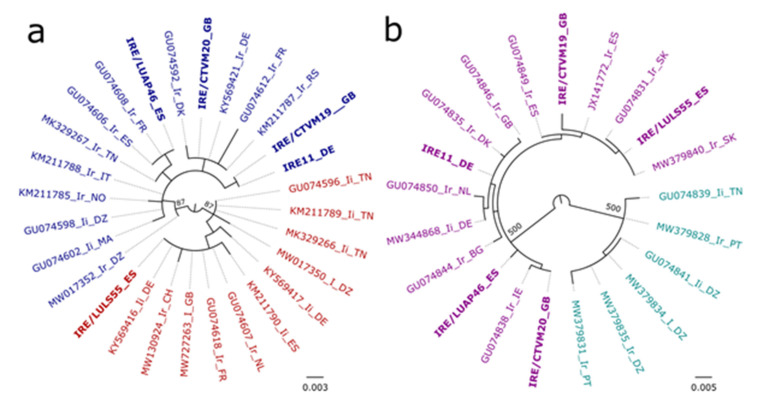
Maximum likelihood phylogenies of 16s rRNA and *trospA* gene fragments amplified from the *Ixodes ricinus* cell lines IRE/LUAP46, IRE/LULS55, IRE/CTVM19, IRE/CTVM20 and IRE11. (**a**) 16s rRNA (441 nucleotide positions): sequences in the 184-AG haplogroup shown in red and sequences in 184-CT haplogroup shown in blue. (**b**) *trospA* (695 nucleotide positions): sequences from North Africa and Portugal shown in turquoise and sequences from Europe, excluding central and southern Portugal, shown in purple. Sequences generated from the *I. ricinus* cell lines are in bold. Sequences obtained from NCBI GenBank are represented by the accession numbers and followed by the country codes. Ir = *I. ricinus*, Ii = *Ixodes inopinatus*, I = *Ixodes* sp. Bootstrap support values greater than 70% are shown adjacent to the nodes. Scale bars represent the number of substitutions per site.

A recent study reported the separation of European *Ixodes* sp. ticks into two “haplogroups” based on the presence of AG or CT bases at position 184/185 in the 16s rRNA gene sequences from ticks reported as *I. ricinus* and *I. inopinatus* [40]. Phylogenetic analysis of the 16S rRNA sequences obtained from the five *I. ricinus* cell lines and selected published sequences from ticks reported as *I. ricinus*, *I. inopinatus* or *Ixodes* sp. (Figure 4a) revealed the clustering of IRE/LULS55 with sequences from ticks belonging to the 184-AG haplogroup (predominantly ticks assigned to *I. inopinatus*) in a single clade (87% support), while sequences from IRE/CTVM19, IRE/CTVM20, IRE/LUAP46 and IRE11 clustered in a separate clade (87% support) with ticks in the 184-CT haplogroup (predominantly ticks assigned to *I. ricinus*). The presence of the AG or CT bases for each haplogroup can be observed clearly in the alignment of the 16s rRNA sequences used in the analysis (Figure S1).

Sequences amplified by the *coxI* PCR from all five cell lines were 100% identical (100% query cover) to between 3 and 15 published sequences from ticks described as *I. ricinus* from Italy, Croatia, Slovakia and Finland, among others. The top eight matches for the cell lines IRE/CTVM19 and IRE11 were identical, but all differed from the top matches for the cell line IRE/CTVM20. The top ten matches for the two Spanish tick cell lines, IRE/LUAP46 and IRE/LULS55, were almost identical to each other, but nearly all different from those for the UK and German tick cell lines. BLASTN analysis limiting to only querying published *I. inopinatus* records (taxid:1538109) showed that *coxI* sequences generated from the *I. ricinus* cell lines were only 98.91% to 99.27% (83% query cover for all matches) identical to sequences derived from ticks assigned in GenBank as *I. inopinatus* from Tunisia, Morocco and Algeria (GU074900, GU074906 and GU074902 [19]). The *coxI* sequences from the *I. ricinus* cell lines also clustered with sequences from ticks assigned in GenBank as *I. ricinus* (94% support) and not with the three *I. inopinatus* sequences in the phylogenetic analysis (Figure S2).

The *trospA* sequences derived from *I. ricinus* cell lines, which were initiated from ticks collected from the UK, Germany and Spain, clustered with sequences derived from ticks assigned in GenBank to *I. ricinus* and *I. inopinatus* from Europe, excluding Central and South Portugal (100% support) (Figure 4b). This is consistent with previous reports of the separation of these ticks into two clades in *trospA* phylogenies: one including specimens from North Africa and Central and South Portugal, and another consisting of specimens from other Eurasian countries [19,41]. Nucleotide differences observed at 18 separate positions in the alignment of the *trospA* sequences in this analysis appeared to be associated with the division into two clades (Figure S3).

### 3.6. Rhipicephalus bursa

Two primary cultures, in L-15/MEM and L-15/L-15B, were initiated from an *R. bursa* egg batch, 20 days after the start of oviposition in 2018. The culture in L-15/L-15B died after 10 months, but the primary culture in L-15/MEM survived and eventually began to proliferate. The first subculture was carried out at 31 months (Table 2) and the cell line RBE/LULS58, established after 35 months, is now at passage 8. Additional primary cultures set up in 2016 (*n* = 1) and 2019 (*n* = 2) using eggs laid by Spanish *R. bursa* ticks are also expected to yield cell lines. RBE/LULS58 comprises a mixture of small, fibroblast-like, relatively non-granular cells growing in spreading clumps, and larger clumps of rounded, sometimes vacuolated, cells (Figure 3c). The 16S rRNA sequence obtained from RBE/LULS58 DNA was 100% identical (94–100% query cover) to sequences from *R. bursa* ticks from Spain (Z97878 [29]) and Turkey (MT302761 [42]) among others.

## 4. Discussion

The establishment of continuous tick cell lines can be a long and challenging process, with a historically low success rate [5,12]. The present study illustrates these points well, representing work conducted over a period of eight years during which the spectrum of success ranged from the *A. reflexus* cell line ARE/LULS41 derived in less than two years from a single primary culture prepared with just nine embryos, to the *H. lusitanicum* lines obtained from three out of nine primary cultures that took 3.5–6.25 years to become established. These four cell lines, and the other new lines derived from *D. reticulatus*, *H. scupense*, *I. ricinus* and *R. bursa*, greatly extend the availability of European tick cell lines and thus the scope of in vitro research that can be conducted in natural pathogen–vector combinations.

European ticks have been reported to harbour a wide range of pathogenic and symbiotic bacteria, including species of the genera *Anaplasma*, *Bartonella*, *Borrelia*, *Coxiella*, *Ehrlichia*, *Francisella*, *Midichloria*, *Neoehrlichia*, *Rickettsia* and *Spiroplasma* [43,44,45,46,47,48]. In some cases, transovarially-transmitted bacteria have hampered efforts to generate embryo-derived cell lines from European ticks, as previously reported for *D. reticulatus*, *Dermacentor marginatus* and *I. ricinus* [49,50]. In the present study, if such bacteria were present in the eggs used to generate our primary cultures, they did not survive and proliferate. The absence of contaminating bacteria means that the new cell lines can be confidently applied in studies on tick-associated human and veterinary pathogens prevalent in Europe, such as *Anaplasma phagocytophilum*, *Bartonella* spp., *Borrelia* spp., *Coxiella burnetii*, *Ehrlichia canis*, *Francisella tularensis*, *Neoehrlichia mikurensis* and *Rickettsia* spp., as well as on symbiotic bacteria, such as *Coxiella*-like and *Francisella*-like endosymbionts and *Spiroplasma*. Further study, including the attempted amplification of additional bacterial genes or whole genome sequencing of the new cell lines, is necessary to determine whether or not any of them harbour bacterial DNA integrated in the tick cell genomes, as reported for *Rickettsia africae* sequences in two *Amblyomma variegatum* cell lines and suspected for *Francisella* sequences in some North American *Dermacentor* spp. cell lines [51,52].

European ticks are also involved in transmission of multiple zoonotic arboviruses of the families Flaviviridae and Reoviridae and the order Bunyavirales, mostly of importance for causing disease in humans [53], with the exception of the livestock pathogen louping ill virus (LIV) in the UK and its close relatives in parts of Europe [54]. Of these, the most widely-distributed are TBEV, transmitted by *I. ricinus* and *I. persulcatus* in Northern, Central and Eastern Europe, and CCHFV transmitted by *Hyalomma* spp. ticks in the Mediterranean and Balkan regions. Several studies on TBEV have used (directly or indirectly) the existing *I. ricinus* cell lines derived from UK and German ticks [7,55,56,57,58,59,60]; the new *I. ricinus* lines derived from Spanish ticks will add a new dimension to research on TBEV–vector interactions. *D. reticulatus* is also suspected to be a natural vector of TBEV [57,61], but in vitro investigations have been hampered by the lack of any cell lines from this species. It will be interesting to compare susceptibility to TBEV of DRE/LULS60 cells and cell lines from the known vector *I. ricinus*, as higher TBEV titres were previously reported in cell lines from *I. ricinus* compared to those from non-vector tick species [55]. To date, in vitro studies on CCHFV have relied on cell lines derived from the Asian vector species *Hyalomma anatolicum* [62,63,64]; the availability of cell lines derived from two European *Hyalomma* spp. will broaden the scope of such studies. Whole genome and RNAseq screening of the new cell lines may also reveal hitherto undescribed novel tick-borne viruses [65], such as the rhabdovirus and iflavirus recently detected in, respectively, the *I. ricinus* cell line IRE/CTVM19 [66] and the *I. scapularis* cell line ISE6 [67,68].

*D. reticulatus*, *I. ricinus* and *R. bursa* are vectors of protozoan parasites of the genus *Babesia* causing disease in, depending on the species, humans and/or domestic animals. Despite several attempts using tropical bovine parasites [69,70], no in vitro culture system for continuous propagation of any *Babesia* sp. in tick cells has yet been developed [10]. As *Babesia* spp. naturally undergo cycles of replication within infected ticks, the potential for development of such a system exists if suitable culture conditions and host cells can be identified. The new tick cell lines reported here could be tested for susceptibility to infection with a range of *Babesia* spp. prevalent in Europe, such as *Babesia canis*, *Babesia divergens* and *Babesia ovis*.

The present study has increased the number of geographic locations within Europe from which ticks, giving rise to cell lines, have originated. Previously, cell lines were available from *I. ricinus* from Germany [11] and the United Kingdom [12], and from *R. sanguineus* from France [13]. Both northern and southern Europe are now better represented, with the new *A. reflexus* and *D. reticulatus* cell lines from Germany and the new *H. lusitanicum*, *H. scupense*, *I. ricinus* and *R. bursa* lines from Spain. The present study depended on the provision of ticks by co-authors and colleagues with access to laboratory colonies and field collection sites, and was therefore biased towards these countries. It is hoped that cell lines can be established in future from ticks originating from additional European regions to provide a more comprehensive coverage of the continent, thereby reflecting potential regional variations in genotype.

This issue is illustrated by the two new *I. ricinus* cell lines derived from Spanish ticks collected at sites in La Rioja approximately 42 km apart. The 16S rRNA genotype of IRE/LUAP46, derived from progeny of a tick feeding in late summer (September), was most similar to archetypal *I. ricinus* sequences from many parts of Europe, and also to the sequences obtained from the cell lines IRE11, IRE/CTVM19 and IRE/CTVM20 derived from northern European ticks. In contrast, the 16S rRNA genotype of IRE/LULS55, derived from progeny of ticks feeding in early spring (February), was most similar to a different subset of *I. ricinus* sequences, as well as some described as *I. inopinatus*. The two species have been reported to be allopatric in Spain [36]; the authors suggested that the distribution area of *I. inopinatus* did not yet reach as far north as La Rioja. An extensive collection of ticks from this province was included in their study, based on 16S rRNA sequences, but they did not find any *I. inopinatus* amongst them [36]. To investigate this further, we examined two additional genes, *coxI* and *trospA*, used in previous studies to differentiate between *I. ricinus* and *I. inopinatus* or to compare ticks from north Africa and Portugal with ticks from the rest of Europe [19,41]. However, these genes did not produce a clear geographic or phylogenetic pattern for the *I. ricinus* cell lines either. Several studies that demonstrate the genetic differentiation of European and North African *I. ricinus* populations [19,71] support the presence of *I. inopinatus* in Southern Europe and North Africa, but call into question the numerous descriptions of this tick species in Central and Northern Europe. As can be observed in the present study and previously [40], the characterisation of a single gene fragment does not guarantee the accurate classification of *I. inopinatus* specimens, and the morphological identification can be challenging. Thus, an extensive study with accurate morphological description of tick samples and deeper genetic characterisation should be performed to investigate the occurrence of *I. inopinatus* across Europe.

While it is outside the scope of the present study to determine whether or not the parent ticks that gave rise to the IRE/LULS55 cell line should be considered a separate species, the availability of cell lines with both archetypal and divergent *I. ricinus* genotypes should help to resolve the issues surrounding the genetic structure of *I. ricinus* populations in Europe and the Mediterranean region [19,36,72]. Even within the group of cell lines displaying the archetypal *I. ricinus* 16S rRNA genotype examined in the present study, the two cell lines IRE/CTVM19 and IRE/CTVM20, derived from the same pool of four egg batches originating from the same location in Southern England, were not identical. These two cell lines were previously found to locate separately in a phylogenetic analysis based on a smaller 16S rRNA gene fragment [37], although sequences from different passage levels of the IRE/CTVM19 cell line maintained in different laboratories still clustered together, suggesting that the differences related to genetic origin rather than from mutations arising during prolonged in vitro cultivation. We also found that the *coxI* and *trospA* sequences from these two sister cell lines each matched most closely with a different set of published sequences.

In conclusion, the ten new tick cell lines reported here will expand the possibilities for studies on isolation, characterisation and control of European ticks and tick-borne microorganisms. ARE/LULS41 is the first continuous cell line derived from any *Argas* species, and joins a small group of argasid tick cell lines [73,74] that can be used to investigate the interactions between soft ticks and the bacterial and viral microorganisms that they harbour [24,74,75]. ARE/LULS41 has a practical advantage over the *Carios capensis* and *Ornithodoros moubata* cell lines reported previously [73,74] in that the cells can be readily cryopreserved and resuscitated. Among the nine new ixodid tick cell lines, those derived from *D. reticulatus*, *H. lusitanicum*, *H. scupense* and *R. bursa* are also the first continuous cell lines to be established and reported from these species. Collectively, all the ixodid cell lines will have wide applications in research on the biology and control of tick-borne diseases of medical and veterinary importance in the European context, including tick-borne encephalitis, Crimean-Congo haemorrhagic fever, tick-borne fever, babesiosis and rickettsioses.

## Figures and Tables

**Table 1:** Geographic origins, dates of collection and host species of engorged female ticks used to provide eggs for cell line generation.

Tick Species	Location (Month and Year of Collection)	Host Species
*Argas reflexus*	Laboratory colony at FLI (September 2015)	Artificial feeding system ^1^
*Dermacentor reticulatus*	Berlin, Germany (November 2018)	Wild boar
*Hyalomma lusitanicum*	Castilla-La Mancha, Spain (February and May 2014)	Deer
*Hyalomma scupense*	Tobía, La Rioja, Spain (February 2016)	Cattle
*Ixodes ricinus*	Tobía, La Rioja, Spain (September 2015) Jubera, La Rioja, Spain (February 2018)	Cattle
*Rhipicephalus bursa*	Jubera, La Rioja, Spain (February 2018)	Cattle

^1^ Ticks were fed on defibrinated swine blood [14].

**Table 2:** Summary of new tick cell lines generated from European tick species in the present study.

Tick Species	Cell Line	Primary Culture (Date)	Culture Medium	1st Successful Passage	Passage Level Reached	1st Successful Cryopreservation (Passage Level)
*Argas reflexus*	ARE/LULS41	December 2015	L-15	11 months	9	23 months (3)
*Dermacentor* *reticulatus*	DRE/LULS60	January 2019	L-15B	15 months	10	29 months (3)
*Hyalomma* *lusitanicum*	HLE/LULS42	March 2014	L-15/L-15B ^1^	14 months	10 ^2^	45 months (3)
	HLE/LULS43	June 2014	L-15/H-lac/L-15B ^1^	14 months	13 ^2^	42 months (4)
	HLE/LULS48	March 2014	L-15/H-lac/L-15B ^1^	26 months	15 ^2^	75 months (12)
*Hyalomma scupense*	HSE/LULS51	April 2016	L-15	26 months	11	54 months (4)
	HSE/LULS59	April 2016	L-15/H-lac ^1^	56 months	9	63 months (5)
*Ixodes ricinus*	IRE/LUAP46	December 2015	L-15	33 months	6 ^2^	45 months (2)
	IRE/LULS55	July 2018	L-15/H-lac/L-15B ^1^	20 months	23	29 months (3)
*Rhipicephalus bursa*	RBE/LULS58	March 2018	L-15/MEM	31 months	8	35 months (3)

^1^ Culture medium prepared using equal volumes of the specified complete media. ^2^ Passage level reached prior to most recent cryopreservation.

## Data Availability

Sequences generated during the course of this study have been deposited in GenBank under accession numbers ON366971–ON366983, ON367517–ON367521 and ON375792–ON375796.

## Supplementary Materials

The following supporting information can be downloaded at: https://www.mdpi.com/article/10.3390/microorganisms10061086/s1. Figure S1: MUSCLE alignment of 16s rRNA gene sequences of *Ixodes ricinus* (Ir) and *Ixodes inopinatus* (Ii) ticks, and *I. ricinus* cell lines; Figure S2: Maximum likelihood phylogeny of *coxI* (1539 nucleotide positions) sequences from *Ixodes ricinus* cell lines, *I. ricinus* ticks and *Ixodes inopinatus* ticks; Figure S3: MUSCLE alignment of *trospA* gene sequences of *Ixodes ricinus* (Ir) and *Ixodes inopinatus* (Ii) ticks, and *I. ricinus* cell lines.
