# Supplementary material for: New Cell Lines Derived from European Tick Species
Source: Microorganisms. 2022 May 25;10(6):1086. doi: 10.3390/microorganisms10061086 (PMC9228755; doi:10.3390/microorganisms10061086)
Supplement: Supplementary file 1 [file microorganisms-10-01086-s001.zip › Bell-Sakyi et al supplementary Figures S1-S3.pptx]

## Slide 1
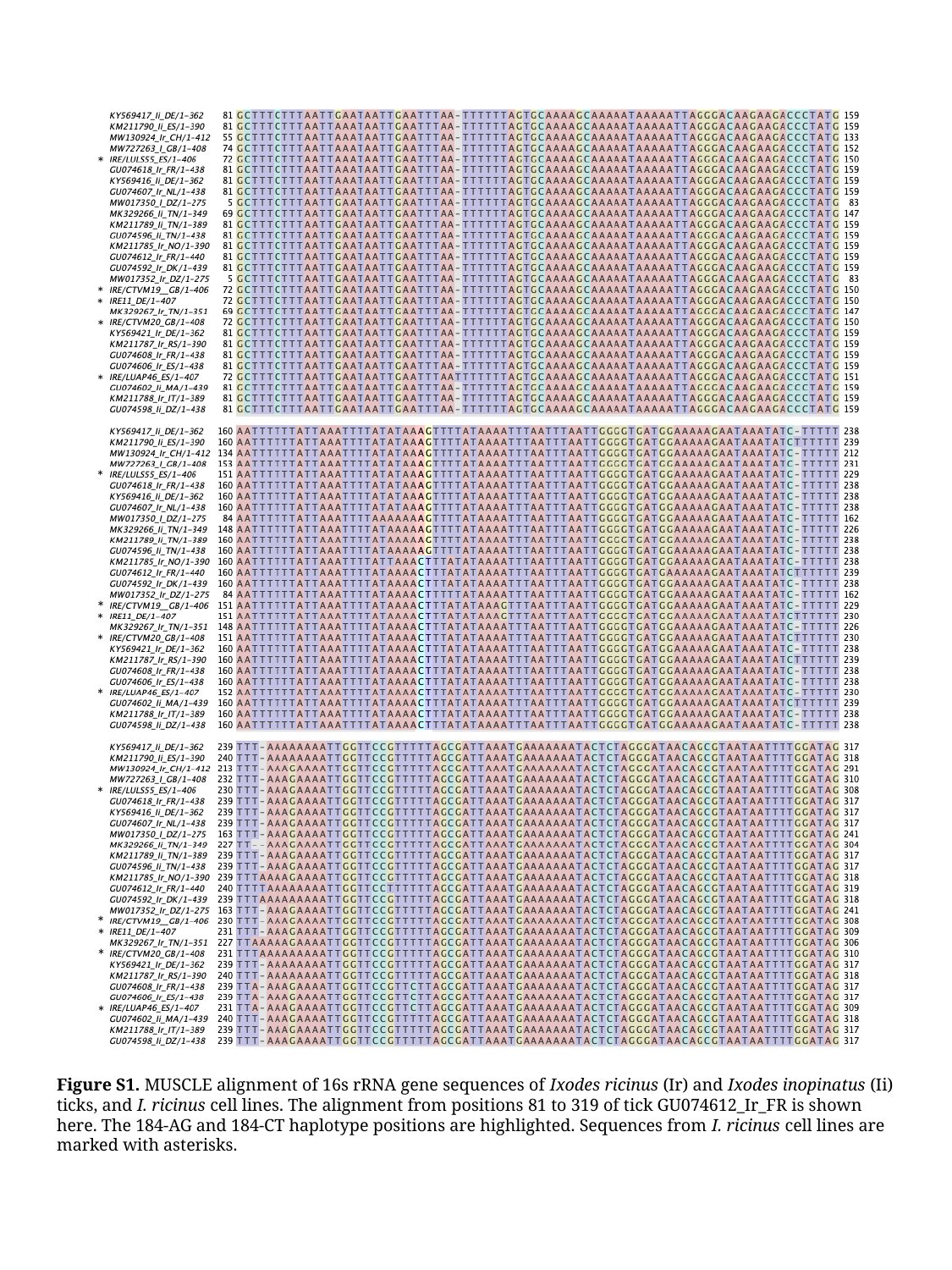

*
*
*
*
*
*
*
*
*
*
*
*
*
*
*
Figure S1. MUSCLE alignment of 16s rRNA gene sequences of Ixodes ricinus (Ir) and Ixodes inopinatus (Ii) ticks, and I. ricinus cell lines. The alignment from positions 81 to 319 of tick GU074612_Ir_FR is shown here. The 184-AG and 184-CT haplotype positions are highlighted. Sequences from I. ricinus cell lines are marked with asterisks.

## Slide 2
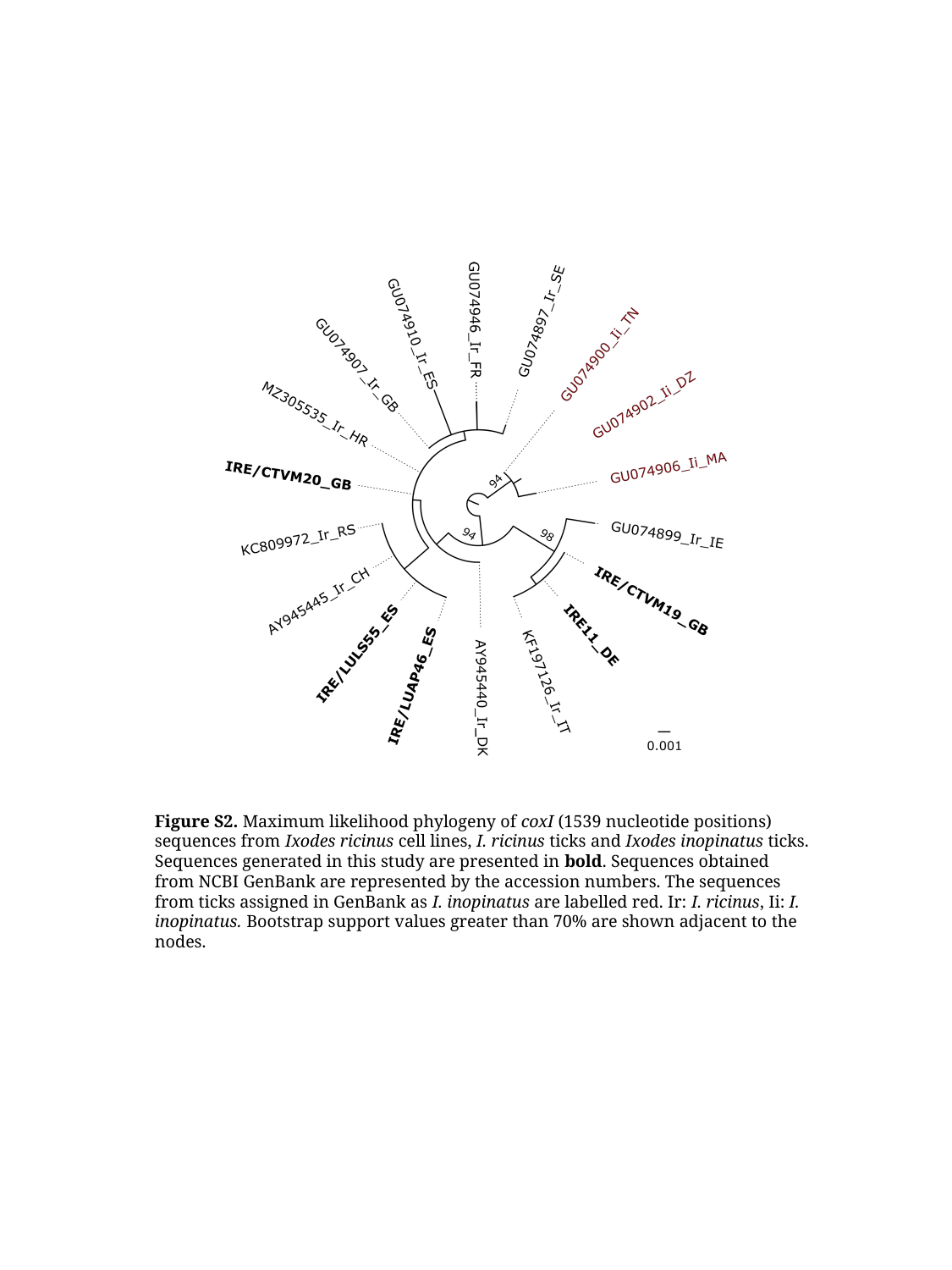

Figure S2. Maximum likelihood phylogeny of coxI (1539 nucleotide positions) sequences from Ixodes ricinus cell lines, I. ricinus ticks and Ixodes inopinatus ticks. Sequences generated in this study are presented in bold. Sequences obtained from NCBI GenBank are represented by the accession numbers. The sequences from ticks assigned in GenBank as I. inopinatus are labelled red. Ir: I. ricinus, Ii: I. inopinatus. Bootstrap support values greater than 70% are shown adjacent to the nodes.

## Slide 3
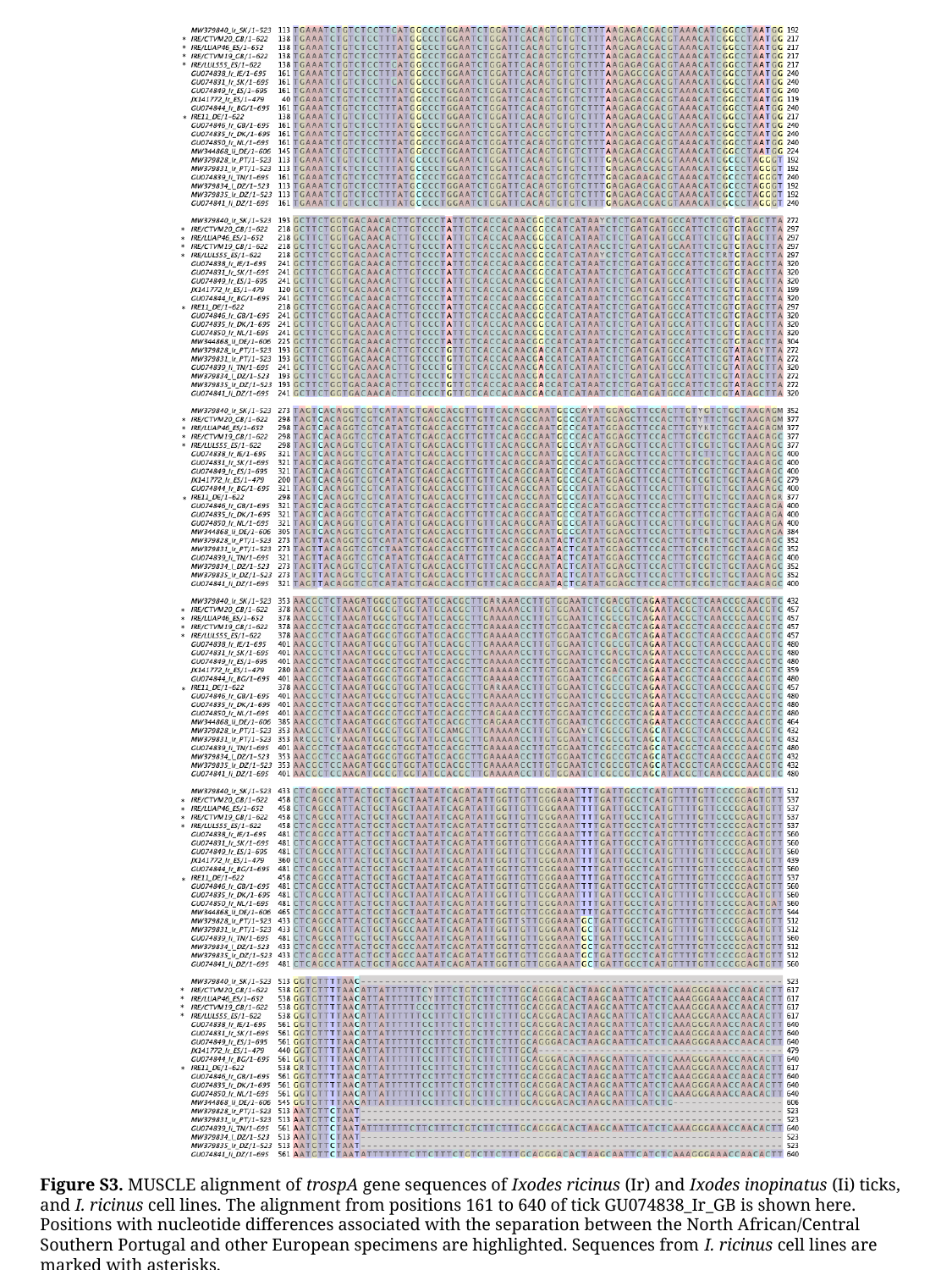

*
*
*
*
*
*
*
*
*
*
*
*
*
*
*
*
*
*
*
*
*
*
*
*
*
*
*
*
*
*
Figure S3. MUSCLE alignment of trospA gene sequences of Ixodes ricinus (Ir) and Ixodes inopinatus (Ii) ticks, and I. ricinus cell lines. The alignment from positions 161 to 640 of tick GU074838_Ir_GB is shown here. Positions with nucleotide differences associated with the separation between the North African/Central Southern Portugal and other European specimens are highlighted. Sequences from I. ricinus cell lines are marked with asterisks.
